# Idiopathic Epididymo‐Orchitis and Pyocele in a Preterm Newborn: A Case Report

**DOI:** 10.1155/crpe/5548695

**Published:** 2026-08-27

**Authors:** Pierrot Sarkis, Fares Chedid, Mohammad Khalil

**Affiliations:** ^1^ Kanad Hospital, Al Ain, Abu Dhabi, UAE

**Keywords:** Epididymo-orchitis, *Escherichia coli* infection, newborn, premature, pyocele

## Abstract

Epididymo‐orchitis (EO) is an extremely rare condition in preterm infants and usually results from hematogenous bacterial spread or urinary infections linked to structural abnormalities. We describe a preterm newborn, born at 36 weeks of gestation, with a left‐sided diaphragmatic hernia. After successful surgery at 12 h of life, the newborn developed red and tender swelling of the right scrotum on the 13th postoperative day. Color Doppler ultrasonography revealed an abrupt cessation of vascular flow at the base of the right scrotum and peri‐testicular fluid. Urinalysis results were unremarkable. Surgical exploration revealed pus in the tunica vaginalis and congestion of the right epididymis and testis, confirming the diagnosis of EO and pyocele. The peri‐testicular space was cleaned and drained. Blood and peri‐testicular pus cultures revealed the growth of *Escherichia coli*, while urine culture showed no growth. After 3 days of scrotal drainage and a 10‐day antibiotic course, the infant improved. Follow‐up ultrasound on Day 14 after surgery showed normal testicular size and structure. EO and pyocele should be considered in acute scrotal swelling in a preterm. The absence of sepsis, urinary tract infection, or urinary tract abnormalities does not preclude the diagnosis.

## 1. Introduction

Epididymitis is a common genitourinary infection in children. Orchitis refers to inflammation of the testis, typically resulting from direct spread from the epididymis, leading to epididymo‐orchitis (EO) [1]. In the neonatal period, EO is uncommon, and most reported cases occur due to sepsis or bacterial infection caused by *Escherichia coli*, *Pseudomonas*, *Salmonella*, or group B streptococcus [2–6]. Hematogenous dissemination and retrograde infection of the epididymis via the patent vas deferens from a urine source are typically regarded as primary pathogenic processes [2, 3, 5, 6]. Urinary tract abnormalities and infections are more frequently associated with EO in infants younger than 1 year and in peri‐pubertal boys [1].

We describe a rare case of right EO with infected peri‐testicular fluid in a preterm newborn without septicemia, urinary tract abnormalities, or concurrent urinary tract infection.

## 2. Case Presentation

A male newborn with a prenatal history of left congenital diaphragmatic hernia was delivered via normal vaginal delivery at 36 weeks of gestation and weighed 2045 g at birth. No birth trauma was mentioned. After the initial stabilization, conventional mechanical ventilation was initiated, and his arterial blood gases remained stable. At 12 h of age, surgery was performed after a multidisciplinary meeting with parents. Primary closure of the left posterior diaphragmatic defect was performed via abdominal surgery. We noticed the presence of a flimsy nonmuscular pleuroperitoneal layer covering the lungs as a sac. The sac was plicated without excision. Antibiotic therapy was initiated. The newborn developed pulmonary hypertension that was treated with vasopressors, and it resolved on postoperative day 4. After the sixth postoperative day, the baby gradually improved and was extubated with a normal chest X‐ray. Blood cultures did not reveal any bacterial growth during or after diaphragmatic surgery. His platelet counts progressively increased from 78,000/dL on Day 3 to 120,000/dL on Day 9 of life. Antibiotics were discontinued on the eighth postoperative day. No wound infection was noticed, and oral feeding was tolerated. He had a normal abdominal examination clinically and had normal stools and weighed 2400 g on Day 12.

On the 13th day of life, right scrotal swelling was noticed during the rounds and was considered initially as a resolving postoperative swelling. The swelling continued to increase for the next 12 h. Urgent surgical consultation noted that the right testis (previously descended in the scrotum) was difficult to palpate in the scrotum, and the right scrotum was inflamed and tender. The left testis (previously undescended) was normal and situated in the inguinal region. Vital signs remained stable throughout, with a white blood cell count of 18,000/μL, of which 62% were polymorphs, and a C‐reactive protein concentration of 127 mg/L. Urinalysis results were unremarkable, and the urine culture performed on the same day revealed no growth.

**FIGURE 1:**
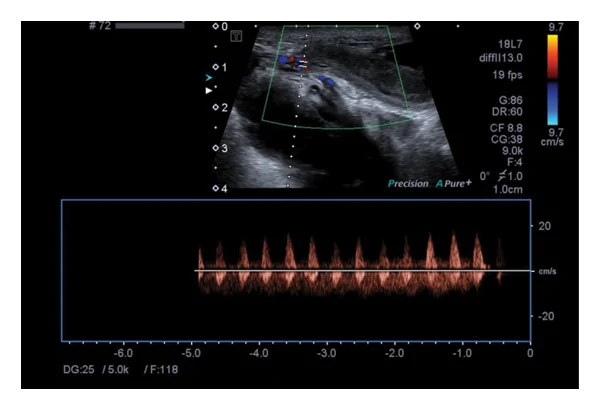
Color Doppler and flow waves proximal to interruption site.

**FIGURE 2:**
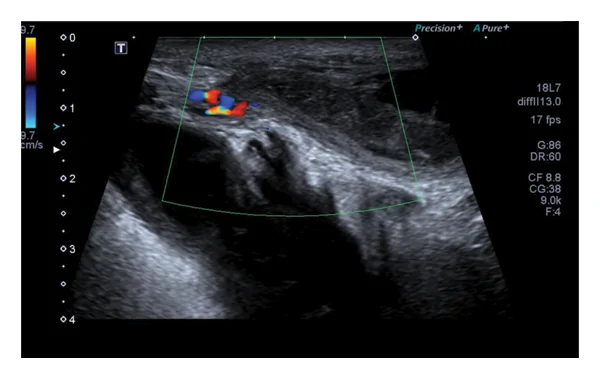
Stopping of flow at inguinal area.

Color Doppler ultrasonography with waves of the inguino‐scrotal area revealed (quote): “Right scrotal sac filled with fluid which shows internal echoes and multiple septations. The fluid is extending into the right inguinal canal. The right inguinal wall is edematous. Vascularity is seen in the inflamed wall of the inguinal region and could be traced until the base of the right scrotum. However, there is abrupt stop of color flow at the base of right scrotum (Figures 1 and 2). Very minimal vascularity is seen in the scrotal wall. An irregular ill‐defined small heterogeneous structure seen in the right lower inguinal canal/upper scrotal canal size 10 × 4 mm. Color Doppler findings could be possible sequelae findings of testicular torsion.”

Antibiotic therapy was initiated. Due to the inability to rule out neonatal testicular torsion, surgical exploration was conducted. Trans‐scrotal incision was preferred because there were no signs of inguinal hernia clinically or on ultrasound, and the right testis was known to be in the scrotum before the event. During scrotal exploration, severe scrotal wall edema, congestion, and redness of the right epididymis and right testis, along with 1–2 mL of pus in the tunica vaginalis were observed. No testicular torsion or abscesses were observed, and neonatal EO and pyocele were confirmed. The peri‐testicular space was then washed and drained. Blood culture and pus culture result from the testicular space showed *E. coli*, and antibiotherapy was adjusted according to antibiogram. After three days, scrotal drain was removed, and antibiotics were withdrawn on the 10th postoperative day, and the baby was discharged home.

**FIGURE 3:**
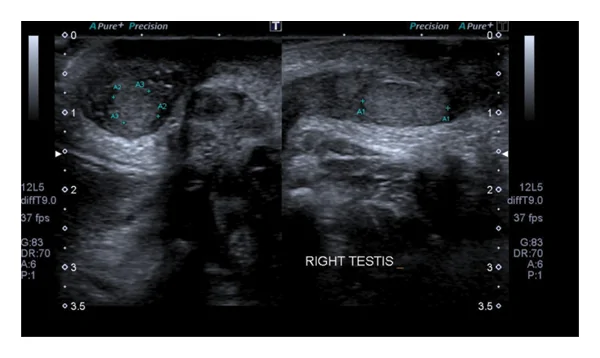
Postoperative ultrasound.

**FIGURE 4:**
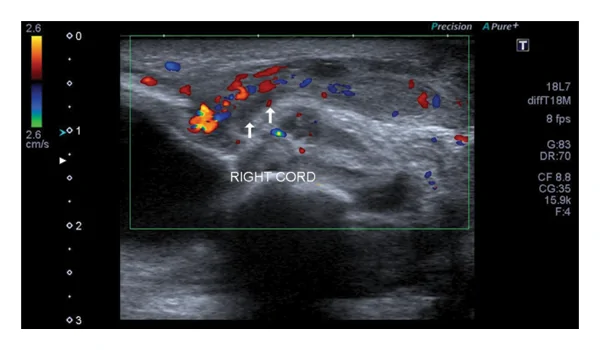
Postoperative color Doppler ultrasound.

During the follow‐up visit in the pediatric surgery unit, color Doppler ultrasonography performed on the 14th postoperative day revealed a normal right testis (11 mm) in the scrotum and a left undescended testis of equal size (11 mm) (Figures 3 and 4).

At 6‐month postoperative follow‐up, the right testis size had normal size and was in scrotal position.

## 3. Discussion

The differential diagnosis of acute isolated scrotal swelling in neonates and preterm infants is challenging. Torsion of the testis continues to be the initial diagnosis to be excluded despite its rarity at this age, because studies on postnatal torsion cases have shown that testes operated on within 12 h were salvaged [7].

Although EO is extremely uncommon in newborns and preterm, it is the second possible diagnosis to be considered in case of acute scrotal swelling. However, differentiation is difficult by clinical examination alone.

Numerous examples of infant EO have been documented in medical literature. The pathogenesis considered in the literature is multiple: secondary to hematogenous dissemination [1–6], ascending urine infection and reflux [1, 6], or a through patent processus vaginalis (from an intra‐abdominal concomitant infection). Our present case in a preterm newborn is different from previously documented examples.

1. In contrast to the majority of documented cases in which EO appeared following hematogenous spread due to sepsis and a preexisting infection [1, 2, 6], our patient exhibited no features of sepsis prior to or throughout the EO episode. Following diaphragmatic hernia repair, the child was normal for a week, without skin infection or abdominal infection. Laboratory results rapidly returned to normal, and blood cultures performed during and after the primary procedure revealed no pathogen growth.
2. Genitourinary abnormalities and urinary tract infections have been frequently related to EO in infants younger than 1 year and at peripubertal age [1, 6, 8]. Aeschimann et al. compared 2 groups with or without previously known urogenital malformation and showed a significant increase in the UTI rate in the group with malformations (3.1% versus 30.8%) [7]. Their study concluded that an existing urogenital malformation is a risk factor for a concomitant UTI during an EO episode. In contrast to previous reports, our case did not develop a urinary tract infection and had no underlying uropathy on ultrasound performed at 13 days of age. According to Chiang et al., following the review of 7 cases of EO in infants less than 3 months of age, a voiding cystogram is indicated in the presence of an ultrasonographic anomaly or a urinary infection [6].
3. The universally accepted treatment of EO is medical treatment with appropriate antibiotics. Surgical exploration becomes mandatory if testicular torsion could not be ruled out by color Doppler ultrasound. In our patient, no blood supply could be identified in the right testis on color Doppler ultrasound, and surgical exploration was indicated. Although color Doppler ultrasound is the main investigation in the differential diagnosis between testicular torsion and EO [6, 9], there is no clear explanation in our case for the interruption of flow on color Doppler ultrasound. The sensitivity of color Doppler ultrasound in the diagnosis of testicular torsion and EO/orchitis in children less than 3 months of age is reported to be 88% and 100%, respectively [6].

The association of peri‐testicular fluid around the affected testis is worth highlighting. All publications that include clinical description of the cases mention the association of hydrocele or pus around the affected testis and the positive culture of the fluid [2, 3, 5, 6]. The pyocele associated with EO is reasonably secondary. However, in the case of an association between EO and pyocele at different degrees of severity, the final diagnosis might be debatable on which pathology is first, EO and/or pyocele.

The diagnosis of intermittent testicular torsion had to be considered because of discordance between color Doppler ultrasonography, which suggested testicular torsion and absence of torsion during surgical exploration. The diagnosis is less likely to be retained for the following reasons. First, the operative finding of congested right epididymis and testis is in favor of EO. Second, the interval between ultrasound and surgery was only 3 h, short lapse of time for the EO to develop following a testicular reperfusion from a torsion–detorsion episode. Moreover, intermittent testicular torsion has not been described during newborn age [10, 11].

The theory of spread through a patent processus vaginalis in the right inguinal canal can be considered in the case presented, especially in association with prematurity and congenital anomalies. In contrast, the absence of intra‐abdominal infection and symptoms during 1 week after recovery from the abdominal repair makes this probability less likely. Moreover, the child did not develop any anomaly of the patent processus vaginalis during the follow‐up period.

Preterm newborn seems at risk of delay in clinical presentation or in diagnosis of EO. In our case, the scrotal swelling was considered abnormal after 12 h of clinical diagnosis. Morris et al. reported an extreme premature of 26 weeks’ gestation admitted with sepsis [5]. At 29 days of age (19 days after ceasing antibiotics), he developed cardiorespiratory instability. Blood culture was positive for *E. coli*. After several days of antibiotic treatment, he had tender swelling of his right scrotum. Ultrasound demonstrated a right scrotal pyocele and testicular hyperemia. Surgical exploration excluded torsion and confirmed an inspissated right pyocele. Chiang et al. reported 2 preterm newborns [6]. A 32‐week preterm infant was admitted at 2 months of age with fever and poor activity, and EO was noted on the third hospital day. A 35‐week preterm infant presented with *Neisseria meningitidis* sepsis, and acute scrotal swelling developed on the seventh hospital day.

Consistent with previous reports, the outcome of the testis in neonatal EO is encouraging. The involved testis of our patient remained normal for 6 months after management.

In summary, neonatal EO is a rare entity but important differential diagnosis to consider in cases of acute scrotal swelling in preterm infants. The absence of sepsis, urinary infection, or urinary tract abnormalities cannot rule out this diagnosis. Surgery is warranted when testicular torsion could not be excluded on color Doppler ultrasound. Pyocele is frequently associated, making the final diagnosis of EO and/or pyocele debatable. Preterm newborns might be at risk of delay in clinical presentation and diagnosis. Reported outcomes are generally favorable, with most cases showing normal testicular appearance and function on a long follow‐up period.

## Author Contributions

Conceptualization/data collection, surgery, and manuscript preparation and editing: Pierrot Sarkis. Neonatal treatment: Fares Chedid and Mohammad Khalil.

Manuscript review and critical appraisal: Pierrot Sarkis, HN, Fares Chedid, and Mohammad Khalil.

## Funding

No funding was received for this manuscript.

## Ethics Statement

Ethical approval was waived by the ethics review committee of the hospital because this was an anonymous case presentation.

## Consent

Informed written consent has been obtained from the parent.

## Conflicts of Interest

The authors declare no conflicts of interest.

## Data Availability

The data that support the findings of this study are available on request from the corresponding author. The data are not publicly available due to privacy or ethical restrictions.
